# Effect of molecular distillation on the anti-inflammatory activity and neurotoxicity of *Asarum* essential oil

**DOI:** 10.3389/fphar.2023.1196137

**Published:** 2023-05-22

**Authors:** Yang Liu, Fang Wang, HuiWen Guo, Dingkun Zhang, Xiaofei Zhang, Zhenfeng Wu, Huiting Li, Yang Xian, Pengfei Yue, Ming Yang

**Affiliations:** ^1^ Key Laboratory of Modern Preparation of TCM, Ministry of Education, Jiangxi University of Chinese Medicine, Nanchang, China; ^2^ College of Chinese Medicine, Jiangxi University of Chinese Medicine, Nanchang, China; ^3^ Chengdu University of Traditional Chinese Medicine, Chengdu, China; ^4^ Shanxi University of Chinese Medicine, Xian, China; ^5^ College of Continuing Education, Jiangxi University of Chinese Medicine, Nanchang, China

**Keywords:** *Asarum* essential oil, molecular distillation, anti-inflammatory activity, neurotoxicity, reducing toxicity and increasing efficacy

## Abstract

*Asarum* essential oil (AEO) has been shown to have good pharmacological activities for the anti-inflammatory and analgesic effects, but increasing the dose may cause toxicity. Therefore, we studied the toxic and pharmacodynamic components of AEO by molecular distillation (MD). Anti-inflammatory activity was assessed using RAW264.7 cells. Neurotoxicity was assessed in PC12 cells and the overall toxicity of AEO was evaluated in the mouse acute toxicity assay. The results showed that AEO is primarily composed of safrole, methyl eugenol, and 3,5-dimethoxytoluene. After MD, three fractions were obtained and contained different proportions of volatile compounds relative to the original oil. The heavy fraction had high concentrations of safrole and methyl eugenol, while the light fraction contained high concentrations of α-pinene and β- pinene. The original oil and all three fractions exhibited anti-inflammatory effects, but the light fraction demonstrated more excellent anti-inflammatory activity than the other fractions. *Asarum* virgin oil and MD products are all neurotoxic. The exposure of PC12 cells to high concentrations of AEO resulted in abnormal nuclei, an increased number of apoptotic cells, increased ROS formation, and decreased SOD levels. Moreover, the results of acute toxicity tests in mice revealed that the light fractions were less toxic than virgin oils and other fractions. In summary, the data suggest that the MD technology enables the enrichment and separation of essential oil components and contributes to the selection of safe concentrations of AEO.

## 1 Introduction

The “Chinese Pharmacopoeia” records that *Asarum* can dispel wind and cold, relieve pain through the orifice, and warm the lungs (National Pharmacopoeia Commission, 2020). It is used for wind chills and colds, headaches, toothache, nasal congestion, rheumatism and pain, phlegm, asthma, and cough. *Asarum* essential oil (AEO) is isolated from the dry rhizome of the Chinese herb *Asarum* [*Asarum heterotropoides Fr. Schmidt var. mandshuricum* (*Maxim.*)*Kitag.*] (Yang et al., 2021), the main components of which are methyl eugenol, safrole, and 3,5-Dimethoxytoluene (Liu et al., 2020). AEO is known for its antipyretic, analgesic, anti-inflammatory, and bacteriostatic properties (Liu et al., 2023). It has been developed into various products which are widely used in clinical medical supplies, pesticides of plant origin, construction materials, and other fields (Maseehullah et al., 2022) (Wu et al., 2020) (Akhlaq et al., 2022).

Multiple studies have documented that AEO exhibits a significant anti-inflammatory effect both *in vivo* and *in vitro* (Choi et al., 2021) (Zhang et al., 2021) (Liu et al., 2022; Liu et al., 2022), which indicates that it is helpful for relieving cough and asthma, relieving bronchospasm, and reducing inflammation in the lungs (Han et al., 2022). Network pharmacological analysis of AEO components showed that methyl eugenol and safrole could act on the inflammatory genes COX-1 and LAT4H (Liu and Wang, 2022). The aforementioned data suggested that methyl eugenol and safrole are the active components of AEO and are may responsible for their anti-inflammatory effects (Fan et al., 2021).

The use of *Asarum* has been documented in “Ben Cao Bie Shuo”, an ancient Chinese pharmacology book written in the Song Dynasty. According to this book, the use of *Asarum* alone should not exceed a dosageof one qian (which is equivalent to 3.72 g). Overdosaging may lead to Qi stuffiness and congestion, ultimately causing death (Hu et al., 2019). Pharmacological studies have confirmed that *Asarum* exhibits a certain degree of toxicity, primarily in the respiratory and nervous systems (Hou et al., 2023). It should be noted that the toxicity of AEO is closely related to the content of volatile oil. The safety of *Asarum* was often ensured through dosage, preparation form, the processing of medicinals, and combination in Chinese medicine. Therefore, a widely used method is to prepare *Asarum* as a decoction, which can reduce its toxicity by prolonging cooking time and decreasing the amount of the volatile oil. However, this limits the medicinal effects of AEO. Furthermore, according to modern drug toxicology studies, the toxicity of *Asarum* mainly originates from aristolochic acid and volatile oil (Antsyshkina et al., 2020). Safrole in volatile oil is the primary toxic component, which can paralyze animal respiratory centers, damage the human nervous system, circulatory system, and respiratory system, and exhibit a carcinogenic effect (Cao et al., 2020) (Kemprai et al., 2020) (Yao et al., 2020). To retain the pharmacological activity of AEO, some researchers separated and removed safrole from the volatile oil by liquid phase preparation, silica gel column chromatography, and adsorption using high-efficiency adsorbent (Nie et al., 2022). However, these methods are costly and, therefore, economically unfeasible for large-scale development and application. Moreover, prior evidence has demonstrated that the toxicity of the whole AEO is higher than that of the enriched safrole oil and methyl eugenol (Liu et al., 2021). The aforementioned results suggest that the combined toxicity of the individual components is less than that of the whole volatile oil, but the underlying reasons for the reduction in toxicity after enrichment remain poorly understood. Hence, further investigation into the components causing toxicity of AEO and the associated mechanisms is necessary to promote safer and more effective market application.

Molecular distillation (MD) is a unique liquid-liquid separation technology that can separate different substances based on their molecular characteristics, such as the mean free path of different molecules under high vacuum conditions (Mahrous and Farag, 2022) (Reza Shirzad Kebria and Rahimpour, 2020). MD can achieve the “refinement” of the main components of volatile oils, “enrichment and enhancement” of the active ingredients, “toxicity reduction” of the toxic components, and “aroma intensification ” (Dantas et al., 2020). In this study, AEO was enriched by MD. The primary components of AEO causing its anti-inflammatory activity, and toxicity were identified and further research was conducted on its toxic/effective components.

## 2 Materials and methods

### 2.1 Reagents and chemicals

n-Hexane (F2124114GC > 99%) was obtained from Aladdin Biotechnology (Shanghai, China). Butyl acetate (RH269812GC ≥ 97.7%) was obtained from Shanghai Yien Chemical Technology Co., Ltd. (Shanghai, China). Anhydrous sodium sulfate (1804081) was procured from Xilong Scientific (Guangdong, China).

Lipopolysaccharide (LPS), dimethyl sulfoxide (DMSO), and dexamethasone (Dex) were purchased from Sigma-Aldrich (St. Louis, MO, United States). Cell culture medium RPMI 1640, DMEM, phosphate buffer saline solution (PBS), and trypsin were obtained from Solarbio Life Sciences (Beijing, China). Fetal bovine serum (FBS) was obtained from Gibco BRL Life Technology (Gibco BRL, Gaithersburg, MD). A cell counting kit 8 (CCK-8) was obtained from Meilunbio (Dalian, China). Interleukin-6 (IL-6) and tumor necrosis factor-α (TNF-α) kits were purchased from Meimian Biotechnology (Jiangsu, China). Superoxide dismutase (SOD) assay kits were procured from Nanjing Jiancheng Bioengineering Institute (Nanjing, China). 4′,6-Diamidino-2-phenylindole dihydrochloride (DAPI) was purchased from Solarbio Life Sciences (Beijing, China). Annexin V-FITC apoptosis detection kit, mitochondrial membrane potential assay kit, and reactive oxygen species (ROS) assay kit were obtained from Beyotime Biotechnology (Shanghai, China).

### 2.2 Plant materials and extraction of AEO

*Asarum* (jk20220118008) was purchased from Chengdu and authenticated as genuine Chinese herbal medicine by Professor Zhang Puzhao of the School of Pharmacy, Jiangxi University of Chinese Medicine. AEO was obtained by steam distillation (*Asarum* was cut into 1 cm, 12 times the amount of water was added, and extracted for 5 h), with an oil yield of approximately 1%. The volatile oil was stored in a refrigerated cabinet at 4°C.

### 2.3 Three-stage molecular distillation of volatile oil

AEO was spread on a thin film on a heated surface to ensure even heating and improve evaporation efficiency and shorten the distillation time. The feed flow rate and scraping speed had a relatively minor impact on the separation results, unlike the evaporation temperature and vacuum degree, which significantly affected the degree of evaporation (Wang et al., 2023). The optimum experimental conditions were determined by pre-experimentation using a short-path MD (F417-9078, UIC GmbH Company, Germany). The optimized conditions were as follows: evaporation temperature of 50°C, vacuum of 8 mbar, primary condensation temperature of 15°C, and secondary condensation temperature of −15°C. Finally, the crude oil (C) was separated, and the fractions were obtained as a heavy fraction (H), a middle fraction (M), and a light fraction (L).

### 2.4 Gas chromatograph-mass spectrometry (GC-MS) analysis of the volatile oil

#### 2.4.1 Preparation of internal standard solution

To a 10-mL volumetric flask, 1,000 μL of butyl acetate was added and the flask was accurately weighed on an analytical balance. The solution was then diluted to 10 mL with n-Hexane and allowed to mix well.

#### 2.4.2 Preparation of sample solution

*Asarum* crude oil was dried with anhydrous Na_2_SO_4_. Next, 50 μL of crude oil and 50 μL of each distillate were pipetted into four 10-mL volumetric flasks. Then, 50 μL of the internal standard solution was added to each flask and diluted to 10 mL with n-hexane. The mixture was evenly mixed and thereafter filtered with a 0.22-μm microporous membrane to obtain the GC sample.

#### 2.4.3 GC conditions

Gas phase conditions: A gas chromatograph-mass spectrometer (7890B-5977A, Agilent Technologies, Inc., United States) and HP-5MS columns were used. The carrier gas was He, the flow rate was 1.0 mL/min, and the split ratio was 10:1. The inlet temperature was 250°C. The heating conditions are shown in Supplementary Table S1.

MS conditions: Electrospray ionization (ESI) was performed. The electron energy was 70 eV, the ion source temperature was 230°C, and the quadrupole temperature was 150°C. Then, the scan range was defined as m/z 30–650 amu, and the injection volume was 0.3 μL. The results were searched with the NIST14. L database in combination with related literature.

### 2.5 Cell lines and treatment

PC12 (Rat adrenal pheochromocytoma) cells were purchased from Shanghai Zhong Qiao Xin Zhou Biotechnology Co., Ltd. (ZQ 0150, Shanghai, China). The cells were cultured in RPMI 1,640 medium containing 1% penicillin/streptomycin and 10% FBS in a 5% CO_2_ incubator at 37°C. The cells were harvested using 0.25% trypsin/EDTA when they reached 80%–90% confluency. The cells were treated with different concentrations of the previously obtained four distillates for the following experiments.

RAW264.7 murine macrophage cells were kindly given by the Research and Experiment Center, College of Traditional Chinese Medicine, Jiangxi University of Chinese Medicine. The cell medium was prepared with DMEM (containing 1% penicillin/streptomycin) and 10% FBS and was cultured in a humidified 5% CO_2_ incubator at 37°C. After the cells reached 80%–90% confluency, they were collected using 0.25% trypsin/EDTA for the subsequent experiments. Then, the cells were treated with 0.5, 0.25, 0.125, and 0.0625 μL/mL concentrations of each of the four distillates.

LPS was diluted to 1 mg/mL in sterile PBS stored at −20°C and then diluted to 100 μg/mL in DMEM medium. Dex was diluted to 4 μg/mL in PBS. LPS and Dex were used in the anti-inflammatory experiment with RAW264.7 cells.

### 2.6 Cell viability assay

PC12 and RAW264.7 cells were harvested by centrifugation and seeded in 96-well plates at 1 × 10^4^ per well. Then, the cells were treated with eight different concentrations (2, 1, 0.5, 0.25, 0.125, 0.0625, 0.03125, and 0.015625 μL/mL) of four distillates (C, H, M, and L) in an incubator for 24 h. According to the kit instructions, RPMI 1,640 containing 10% CCK-8 reagent was added to each well. After incubation at 37°C for 2 h, the optical density (OD) value of each well was measured at 450 nm using a microplate reader (Multiskan GO, Thermo Fisher Scientific, United States).

### 2.7 Measurement of anti-inflammatory activity

A total of 4 × 10^4^ RAW264.7 cells per well were cultured in 24-well plates. Four concentrations (0.5, 0.25, 0.125, and 0.0625 μL/mL) of distillates C, H, M, and L were added for intervention on the second day. After 2 h of incubation, the cells were induced by LPS (100 μg/mL). The untreated RAW264.7 cells served as the control group, the cells induced by LPS without dosing were used as the model group, and dexamethasone was added for the Dex group. After 24 h of incubation, cell supernatants were collected for further experiments with commercial IL-6 and TNF-α kits. Then, after rinsing the plate five times, the kit reagents were added to the reaction plate and incubated at 37°C for 30 min. After washing the samples five times, reagents were added following the manufacturer’s instructions and reacted for 10 min. The OD values of the wells were detected at 450 nm via a microplate reader, and the anti-inflammatory activity of the cells was calculated.

### 2.8 Measurement of the oxidative enzyme system

PC12 cells were collected by trypsin and seeded in 6-well plates with 4 × 10^5^ cells per well. The cells were cultured overnight and exposed to 1, 0.5, 0.25, and 0.125 μL/mL of the four distillates (C, H, M, and L). Then, the cells were incubated in an incubator for 24 h. The cells and cell supernatants were centrifuged and collected in 2 mL EP tubes. Thereafter, the cells were broken using a cell disruptor and stored at −80°C for subsequent assays for the determination of oxidative enzymes. The SOD levels in PC12 cells were measured using the 96-well plates in the commercial assay kits with five replicate wells per sample. The OD values were measured via a microplate reader.

### 2.9 DAPI and annexin V-fluorescein isothiocyanate (FITC)/propidium iodide (PI) staining

DAPI and Annexin V-FITC were used to detect the apoptosis of PC12 cells. For DAPI staining, 4 × 10^5^ PC12 cells were cultured in each well of the 6-well plates. Four different concentrations (1, 0.5, 0.25, and 0.125 μL/mL) of distillates C, H, M, and L were separately added to the cells and incubated for 24 h. After incubation, the cell supernatants were discarded. The cells were washed twice in PBS, and fixed with 4% paraformaldehyde (PFA) (DF0135, Leagene Biotechnology, China) for 15 min. Thereafter, the DAPI reagent was added for 5–10 min at room temperature (RT), followed by washing with PBS twice. The stained cells were observed under a fluorescence microscope (Nikon, Japan).

Annexin V-FITC assay was performed according to the manufacturer’s instructions. PC12 cells were collected after trypsin digestion and centrifuged (1,000 rpm, 5 min). After resuspending with PBS, the cells were centrifuged again, and the supernatants were discarded. Next, 195 μL Annexin V-FITC binding reagent was added. The cells were resuspended and 5 μL of Annexin V-FITC and 10 μL of PI staining solution were added. The samples were incubated for 10–20 min at RT under a dark condition. A flow cytometer (Becton, Dickinson, and Company, United States) was used to analyze the stained cells.

### 2.10 Measurement of ROS levels

The ROS levels in PC12 cells were determined using the fluorescent probe DCFH-DA. Approximately 4 × 10^5^ cells were seeded in each well of a 6-well plate, and the ROS level was measured after treatment with the distillates (C, H, M, and L) for 24 h. To each well, 2 mL of DCFH-DA was added, followed by incubation at 37°C for 20 min. Thereafter, the cells were rinsed thrice with serum-free RPMI 1,640 medium. The cells were then studied using a flow cytometer with an excitation wavelength of 488 nm and an emission wavelength of 525 nm.

### 2.11 Measurement of mitochondrial membrane potential

JC-1 was used as a fluorescent probe in the Mitochondrial Membrane Potential Assay Kit (JC-1) for early apoptosis detection. PC12 cells (4 × 10^5^ per well) were seeded in 6-well plates and incubated with four concentrations (1, 0.5, 0.25, and 0.125 μL/mL) of the four distillates (C, H, M, and L) for dosing intervention. After 24 h of intervention, the experiment was performed according to the manufacturer’s instructions. JC-1 staining solution was added to the cells and cultured at 37°C for 20 min. A fluorescence microscope was used to observe the fluorescence of the cells.

### 2.12 Effect of AEO on acute toxicity in mice

A total of 90 SPF KM mice (6–8 weeks old; 18–22 g each), half male and half female, were obtained from the Experimental Animal Center of the Jiangxi University of Chinese Medicine. This experiment was approved by the Animal Ethics Committee of the university (Animal Ethics Number: 20220306029) and strictly followed the Guidelines for the Management and Use of Laboratory Animals. The acute toxicity test was performed to assess the toxicity of AEO, and the *LD*_*50*_ value was used as the assessment index. The mice were divided into 22 groups: blank (saline), control (20% Tween-80), and 20 experimental groups (four distillates, five dose groups for each distillate, six mice in each group, half male and half female). According to the pre-experimental results, the doses of both crude oil and H distillate were 1.0792, 1.3489, 1.6862, 2.1077, and 2.6347 g/Kg; the concentrations of M were 2.1077, 2.6347, 3.2865, 4.1080, 5.1349 g/Kg, and the administration concentrations of L distillate were 2.6347, 3.2865, 4.1080, 5.1349, 6.4185 g/Kg. The mice fasted without water for 12 h before the experiment. After 12 h, the four distillates were administered by gavage at 0.2 mL/10 g per mouse.

### 2.13 Statistics and analysis

Data were processed using SPSS 21.0 (IBM, New York, NY, United States) for plotting and statistical analysis. Graphs were constructed using GraphPad Prism 9.0 (GraphPad Software, CA, United States) and SIMCA 14.1 (Umetrics, Sweden). Data were expressed as mean ± standard deviation (‾X ± SD) and compared using one-way analysis of variance (ANOVA). *p* < 0.05 was considered statistically significant.

## 3 Results

### 3.1 GC-MS analysis of different distillates

**FIGURE 1:**
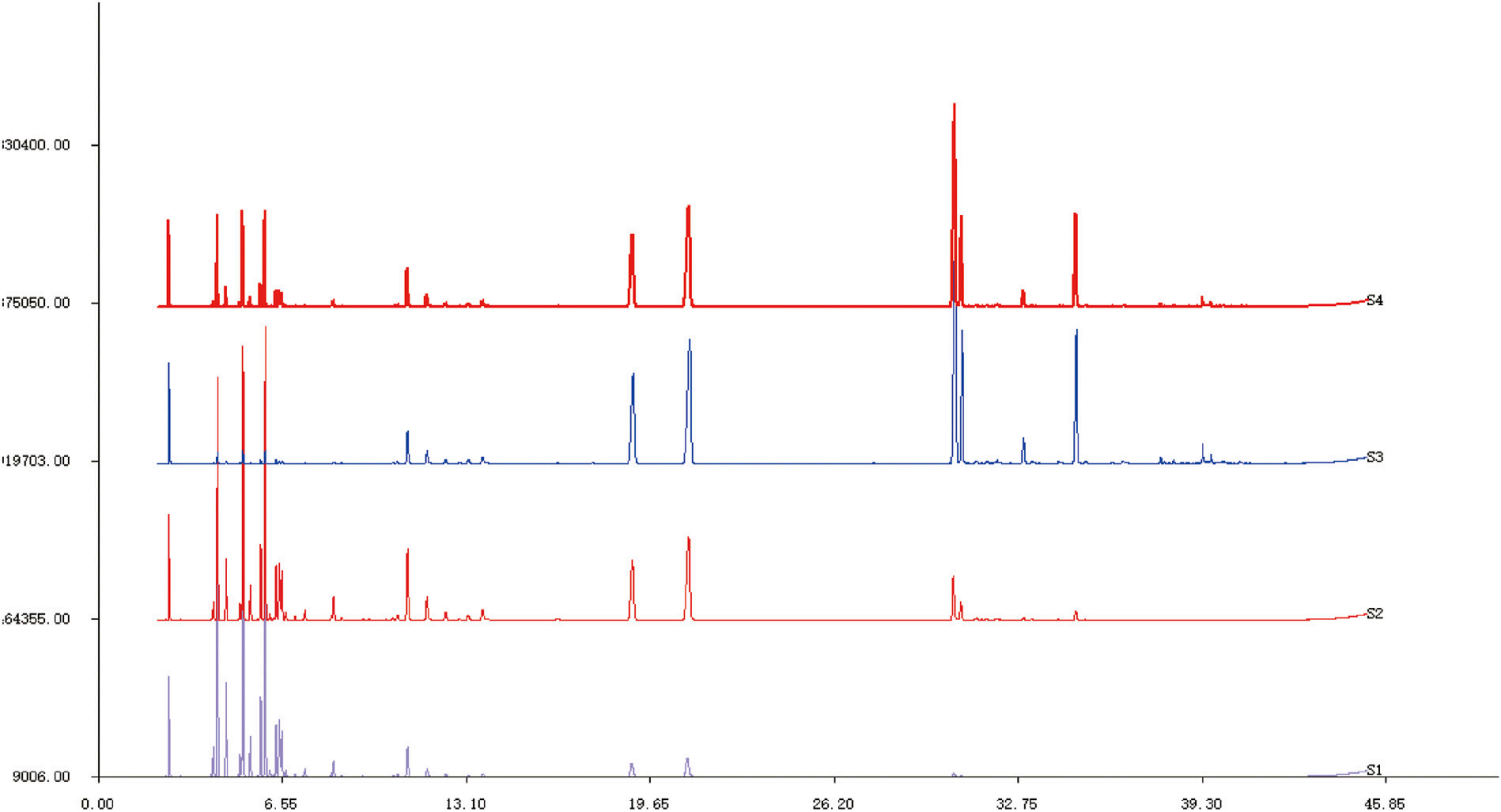
GC-MS spectra of *Asarum* volatile crude oil and each distillate after molecular distillation S1: Light distillate (L). S2: Medium distillate (M). S3: Heavy distillate (H). S4: *Asarum* volatile crude oil (C).

*Asarum* volatile crude oil (C) was divided into three distillates, heavy oil (H), medium oil (M), and light oil (L) via MD. A total of 34 components were identified and isolated from the *Asarum* crude oil, among which methyl eugenol had the highest content (24.02%), and the others were mainly safrole (17.08%), 3,5-dimethoxytoluene (10.62%), myristyl ether (8.20%), 3,4,5-trimethoxytoluene (7.04%), 3-carene (4.77%), β-pinene (4.51%), eugenolone (3.00%), lobenol (1.21%), and levulinic-limonene (1.05%) (Figure 1, S4). The primary components of medium oil and light oil were also analyzed through distillation. The results showed that the main components of distillates M and L were (1S) -(−)-alpha-Pinene (10.64%, 19.84%), β-pinenelaevo (12.51%, 19.69%), and (1S) -(+)-3-Carene (13.96%, 19.63%). Unlike the light fraction, the M fraction contained safrole (12.55%) and 3,5-dimethoxytoluene (7.93%). The main components of the heavy fraction were methyleugenol (31.36%), safrole (19.42%), and 3,5-dimethoxytoluene (12.01%). From the aforementioned results, it is evident that the components in different distillates have significant differences in mass fraction. Therefore, by using MD, the separation and enrichment of AEO was achieved (Table 1).

**TABLE 1:** Chemical composition and content of each distillate after molecular distillation.

No.	RT	CAS	Common name	Quality fraction (%)			
				C	H	M	L
1	2.4974	000123-86-4	Butyl acetate	3.8947	4.0919	4.5102	4.9352
2	4.0801	002867-05-2	alpha-Thujene	0.2791	—	—	1.4746
3	4.2314	007785-26-4	(1S)-(−)-alpha-Pinene	4.183	0.465	10.6367	19.8361
4	4.5339	000079-92-5	Camphene	0.9851	—	2.6924	4.7972
5	5.0363	028634-89-1	Bicyclo [3.1.0]hex-2-ene,4-	0.2417	—	0.7651	—
6	5.1281	000127-91-3	beta-Pinene	4.5104	0.5572	12.505	19.6851
7	5.3982	000123-35-3	Myrcene	0.5025	—	1.6231	2.1255
8	5.771	000099-83-2	α-Phellandrene	1.1322	0.1483	3.522	4.3413
9	5.9276	000498-15-7	(1S)-(+)-3-Carene	4.7743	0.6086	13.9615	19.6277
10	6.3111	000527-84-4	o-Cymene	0.8599	0.1629	0.1143	2.9636
11	6.4354	000138-86-3	(1S)-Limonene	1.0493	—	3.3502	3.9054
12	6.5164	000470-82-6	1,8-Cineole	0.791	0.1553	2.4182	2.6329
13	6.6677	003779-61-1	trans-β-Ocimene	0.1107	—	0.3678	0.3693
14	7.3483	000099-85-4	gamma-Terpinene	0.17	—	0.5435	0.3142
15	8.3585	000586-62-9	Terpinolene	0.4575	—	1.4383	0.2107
16	10.6327	000076-22-2	(+/−)-Camphor	0.2077	—	—	—
17	10.9893	000503-93-5	Eucarvone	3.001	2.3242	5.3055	2.4981
18	11.6807	000507-70-0	Borneol	1.2083	1.0079	—	0.7308
19	12.3506	000562-74-3	4-Carvomenthenol	0.3645	—	0.6204	0.3645
20	13.1609	000098-55-5	α-Terpineol	0.407	—	—	—
21	13.6632	000140-67-0	Estragole	0.6339	0.5721	0.9396	0.3029
22	19.0166	004179-19-5	3,5-Dimethoxytoluene	10.6157	12.0113	7.9303	1.9804
23	21.0207	000094-59-7	Safrole	17.0851	19.4187	12.5543	3.15
24	30.4796	000093-15-2	Methyleugenol	24.0203	31.3644	4.0635	0.3947
25	30.7227	006443-69-2	3,4,5-Trimethoxytoluene	7.0425	9.1769	1.3602	—
26	31.2629	017334-55-3	Calarene	0.2472	0.2764	0.2097	—
27	31.9867	036577-33-0	(−)-Guaia-6,9-Diene	0.1855	0.4505	—	—
28	32.9321	000607-91-0	Myristicin	1.5114	2.0284	0.2217	—
29	33.2292	054274-73-6	1-Epibicyclosequiphellandren	0.1409	—	—	—
30	34.7957	000607-91-0	Myristicin	8.1986	11.1031	0.7939	—
31	37.81	000487-11-6	Elemicin	0.2202	0.3604	—	—
32	38.2746	005353-15-1	γ-Asarone	0.1092	0.1725	—	—
33	39.3064	018607-90-4	Kakuol	0.5873	0.792	—	—
34	39.6035	005986-55-0	patchouli alcohol	0.2721	0.3147	—	—
Monoterpene hydrocarbons				13.5813	1.3141	53.7151	77.2481
Sesquiterpene hydrocarbons				0.4327	1.2153	0.2097	—
Monoterpenoids				1.9798	1.0079	0.6204	1.0953
Monoterpene ketone				3.5883	3.5136	5.7304	2.7235
Ether				69.4369	86.2078	27.8635	5.828
% Identified of total peaks area				89.019	93.2587	88.1391	86.8949

From these results, the contents of monoterpenes in crude oil, H, M, and L were 13.58, 1.31, 53.72, and 77.25%, respectively, whereas the contents of ethers in C, H, M, and L were 69.44, 86.21, 27.86, and 5.83%, respectively. The monoterpenes, with boiling points generally in the range of 140°C–180°C, exhibit analgesic, antibacterial, detoxification, and diuretic effects. For example, the boiling point of β-pinenelaevo is 155°C–156°C, and (+) -3-caren has a boiling point of 168°C–169°C. However, the ethers in AEO had higher boiling points, such as safrole 232°C–234°C and methyl eugenol 254°C–255°C. These ether components exhibited neuroleptic effects and were also the primary toxic components of AEO. From these results, it can be concluded that the light distillate is enriched in low-boiling-point components, while the heavy distillate is enriched in high-boiling-point components.

### 3.2 Cell viability of RAW264.7 cells and PC12 cells

To investigate the safety concentrations of the four distillates C, H, M, and L on RAW264.7 cells and PC12 cells, eight different concentrations (2, 1, 0.5, 0.25, 0.125, 0.0625, 0.03125, and 0.015625 μL/mL) of these distillates were added to the RAW264.7 cells and PC12 cells to detect cell viability for 24 h.

**FIGURE 2:**
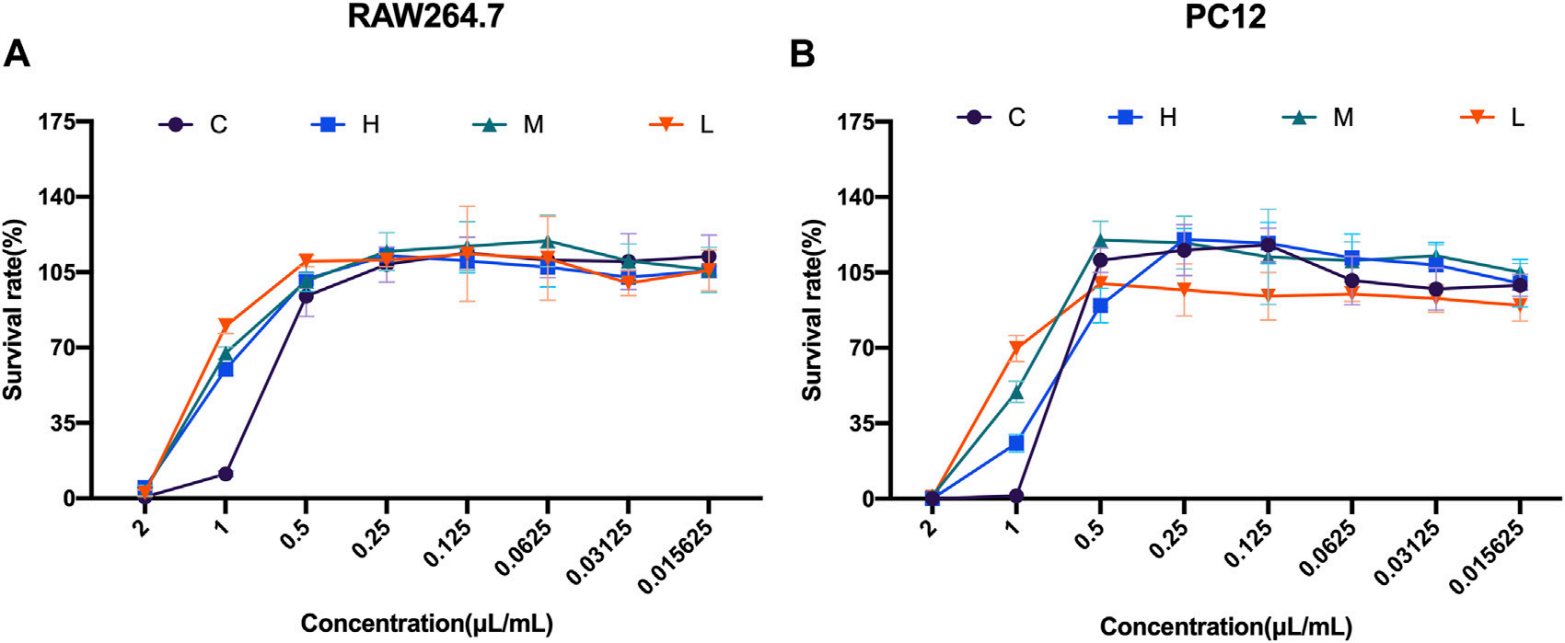
Cell viability studies of *Asarum* volatile oil and its distillates with RAW264.7 cells and PC12 cells **(A)** Cell viability on RAW264.7 cells, with eight concentrations (2, 1, 0.5, 0.25, 0.125, 0.0625, 0.03125, and 0.015625 μL/mL) of C (*Asarum* volatile crude oil), H (heavy distillate), M (medium distillate), and L (light distillate). **(B)** PC12 cells were treated with eight concentrations (2, 1, 0.5, 0.25, 0.125, 0.0625, 0.03125, and 0.015625 μL/mL) of C (*Asarum* volatile crude oil), H (heavy distillate), M (medium distillate), and L (light distillate) to measure cell viability.

The cell viability results of RAW264.7 cells showed that the survival rate was close to 0 at a concentration of 2 μL/mL. However, the survival rates increased with the decrease in the distillate concentration for all the distillates. Especially at 1 μL/mL concentrations, the cell viability with heavy, medium, and light distillates was significantly higher than that with crude oil. Meanwhile, when the concentrations of crude oil, heavy distillate, medium distillate, and light distillate were all less than or equal to 0.5 μL/mL, the cell viability of RAW264.7 cells was greater than 85%. Therefore, four concentrations (0.5, 0.25, 0.125, and 0.0625 μL/mL) of C, H, M, and L were selected for the following experiments on RAW264.7 cells (Figure 2A).

Furthermore, the cell viability results from PC12 cells showed an increase in survival rate with the decrease of concentrations of C, H, M, and L. At 2 μL/mL concentration of the four distillates, the survival rates of PC12 cells were almost 0, while at a concentration of 1 μL/mL of C, H, M, and L, the cell viability of the C group was lower than that of the H, M, and L groups. Notably, the survival rates of PC12 cells were greater than 85% at a concentration of 0.5 μL/mL for all four distillates. This indicates that 0.5 μL/mL was a safe distillate concentration for PC12 cells (Figure 2B).

### 3.3 IL-6 levels in RAW264.7 cells after treatment with AEO and its different distillates

**FIGURE 3:**
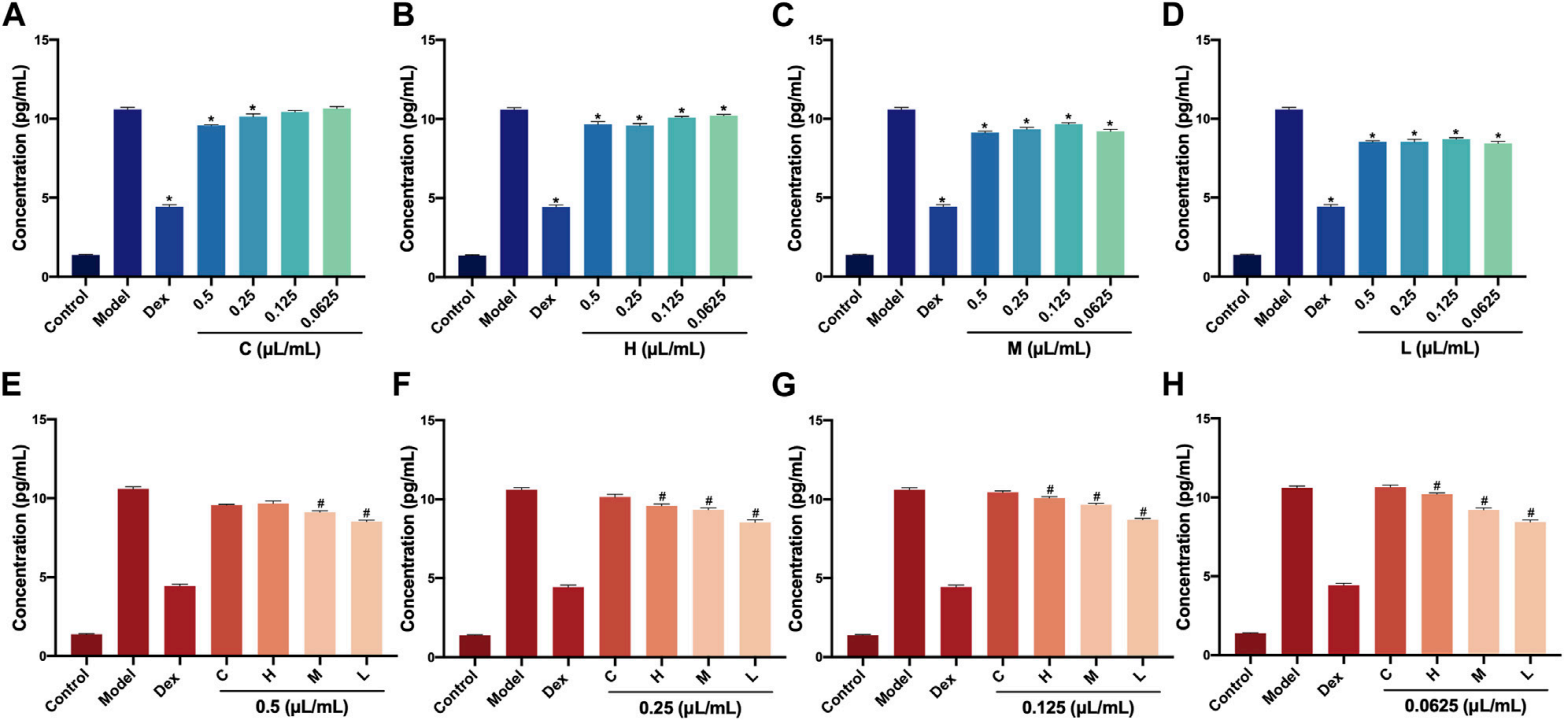
Levels of IL-6 in RAW264.7 cells after treatment with *Asarum* volatile crude oil and different distillates **(A)** Concentrations of IL-6 in RAW264.7 cells with 0.5, 0.25, 0.125, and 0.0625 μL/mL of **(C)**. **(B)** Concentrations of IL-6 in RAW264.7 cells with 0.5, 0.25, 0.125, and 0.0625 μL/mL of **(H)**. **(C)** Concentrations of IL-6 in RAW264.7 cells with 0.5, 0.25, 0.125, and 0.0625 μL/mL of M. **(D)** Concentrations of IL-6 in RAW264.7 cells with 0.5, 0.25, 0.125, and 0.0625 μL/mL) of L. **(E)** Concentrations of IL-6 in RAW264.7 cells after treatment at 0.5 μL/mL of C, H, M, and L. **(F)** Concentrations of IL-6 in RAW264.7 cells after treatment at 0.25 μL/mL of C, H, M, and L. **(G)** Concentrations of IL-6 in RAW264.7 cells after treatment at 0.125 μL/mL of C, H, M, and L. **(H)** Concentrations of IL-6 in RAW264.7 cells after treatment at 0.0625 μL/mL of C, H, M, and L. C (*Asarum* volatile crude oil), H (heavy distillate), M (medium distillate), L (light distillate).*p < 0.05, compared with model group, #*p* < 0.05, compared with the C group.

The IL-6 level in RAW264.7 cells was determined via the commercial ELISA kit after treatment with AEO and its different distillates. First, the IL-6 levels at different concentrations (0.5, 0.25, 0.125, and 0.0625 μL/mL) of crude oil, heavy distillate, medium distillate, and light distillate were compared. The results showed that compared with the LPS-induced model group, there were decreasing trends in IL-6 levels in the Dex groups and four concentration groups of H, M, and L. Furthermore, the IL-6 levels in 0.5 and 0.25 μL/mL groups of C were decreased (*p* < 0.05, vs. model group). These results indicated that distillates H, M, and L, obtained from AEP by MD, could reduce the contents of pro-inflammatory cytokine IL-6 compared with that of the model group (Figures 3A–D).

Then, the levels of IL-6 in C, H, M, and L were analyzed at the concentration (0.5, 0.25, 0.125, and 0.0625 μL/mL)of distillates. Compared with the C group, the IL-6 levels were significantly lower in M and L groups for 0.5 L/mL distillate concentration (*p* < 0.05). When the concentrations were 0.25, 0.12,5, and 0.0625 μL/mL, the IL-6 levels IL-6 in H, M, and L groups were clearly decreased (*p* < 0.05, vs. C group). This indicated that distillates H, M, and L showed a relative decrease in the content of pro-inflammatory cytokine IL-6 at the same concentrations compared to crude oil in a dose-dependent manner (Figures 3E–H).

### 3.4 TNF-α levels in RAW264.7 cells after treatment with AEO and its different distillates

**FIGURE 4:**
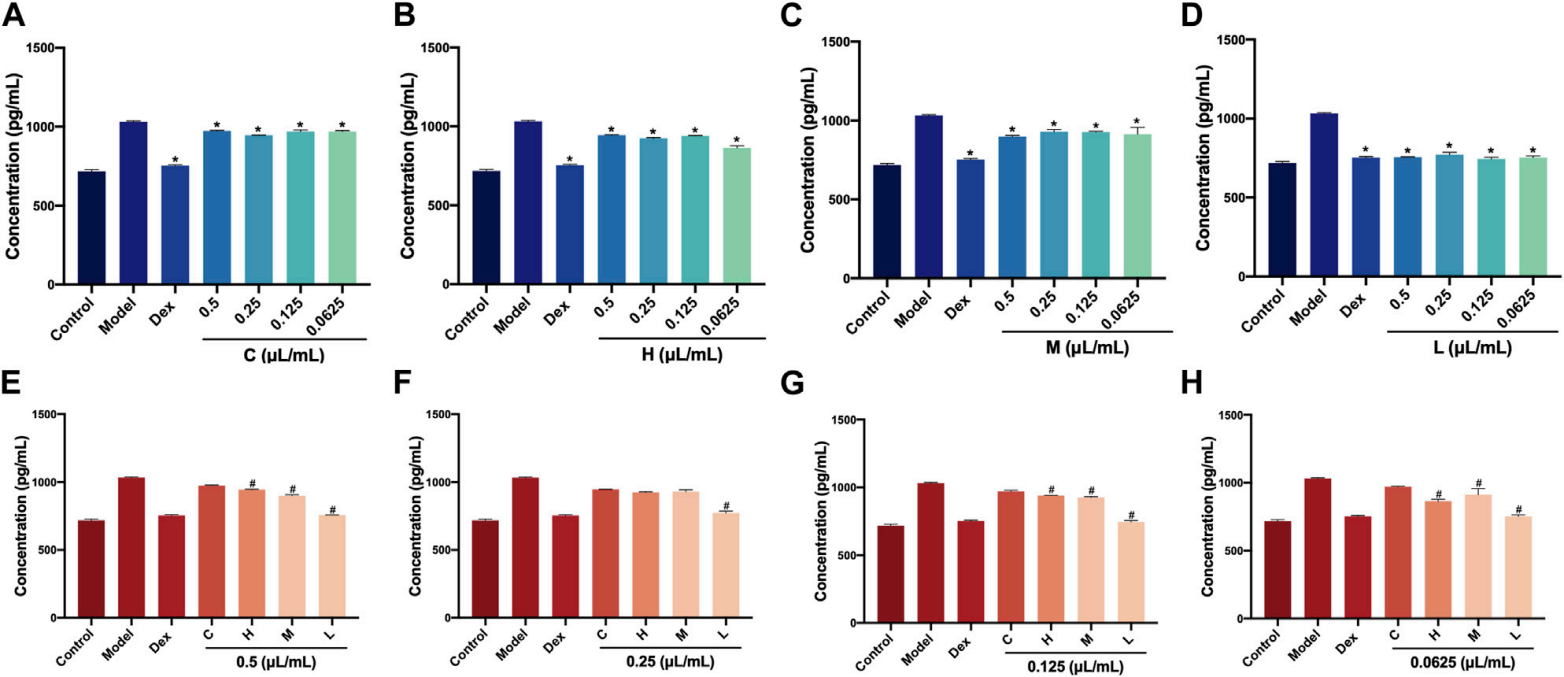
Levels of TNF-α in RAW264.7 cells after treatment with *Asarum* volatile crude oil and different distillates **(A)** Concentrations of TNF-α in RAW264.7 cells with 0.5, 0.25, 0.125, and 0.0625 μL/mL of **(C)**. **(B)** Concentrations of TNF-α in RAW264.7 cells with 0.5, 0.25, 0.125, and 0.0625 μL/mL of **(H)**. **(C)** Concentrations of TNF-α in RAW264.7 cells with 0.5, 0.25, 0.125, and 0.0625 μL/mL of M. **(D)** Concentrations of TNF-α in RAW264.7 cells with 0.5, 0.25, 0.125, and 0.0625 μL/mL) of L. **(E)** Concentrations of TNF-α in RAW264.7 cells after treatment at 0.5 μL/mL of C, H, M, and L. **(F)** Concentrations of TNF-α in RAW264.7 cells after treatment at 0.25 μL/mL of C, H, M, and L. **(G)** Concentrations of TNF-α in RAW264.7 cells after treatment at 0.125 μL/mL of C, H, M, and L. **(H)** Concentrations of TNF-α in RAW264.7 cells after treatment at 0.0625 μL/mL of C, H, M, and L. C (*Asarum* volatile crude oil), H (heavy distillate), M (medium distillate), L (light distillate).*p < 0.05, compared with model group, #*p* < 0.05, compared with C group.

AEO also exhibits pro-inflammatory effects. Therefore, we investigated the TNF-α levels in RAW264.7 cells after AEO, and H, M, and L intervention. Similarly, the TNF-α levels in the four concentrations of C, H, M, and L were measured first. Compared with the LPS-induced model group, the TNF-α levels in the Dex groups and four concentrations (0.5, 0.25, 0.125, and 0.0625 μL/mL) of the H, M, and L groups showed a decreasing trend (*p* < 0.05) (Figures 4A–D). Further, the TNF-α levels in C, H, M, and L at the same concentration were compared. The results showed that at 0.5, 0.125, and 0.0625 μL/mL, the TNF-α levels in H, M, and L were notably lower than that of C group. Only the TNF-α level in the L group was decreased (*p* < 0.05, vs. C group) when the concentration was 0.25 μL/mL (Figures 4E–H). These results illustrated that the three distillates (H, M, and L) could reduce the release of TNF-α in RAW264.7 cells and exert anti-inflammatory effects compared with crude oil.

### 3.5 Orthogonal partial least squares-discriminant analysis (OPLS-DA) of the anti-inflammatory activity of AEO

**FIGURE 5:**
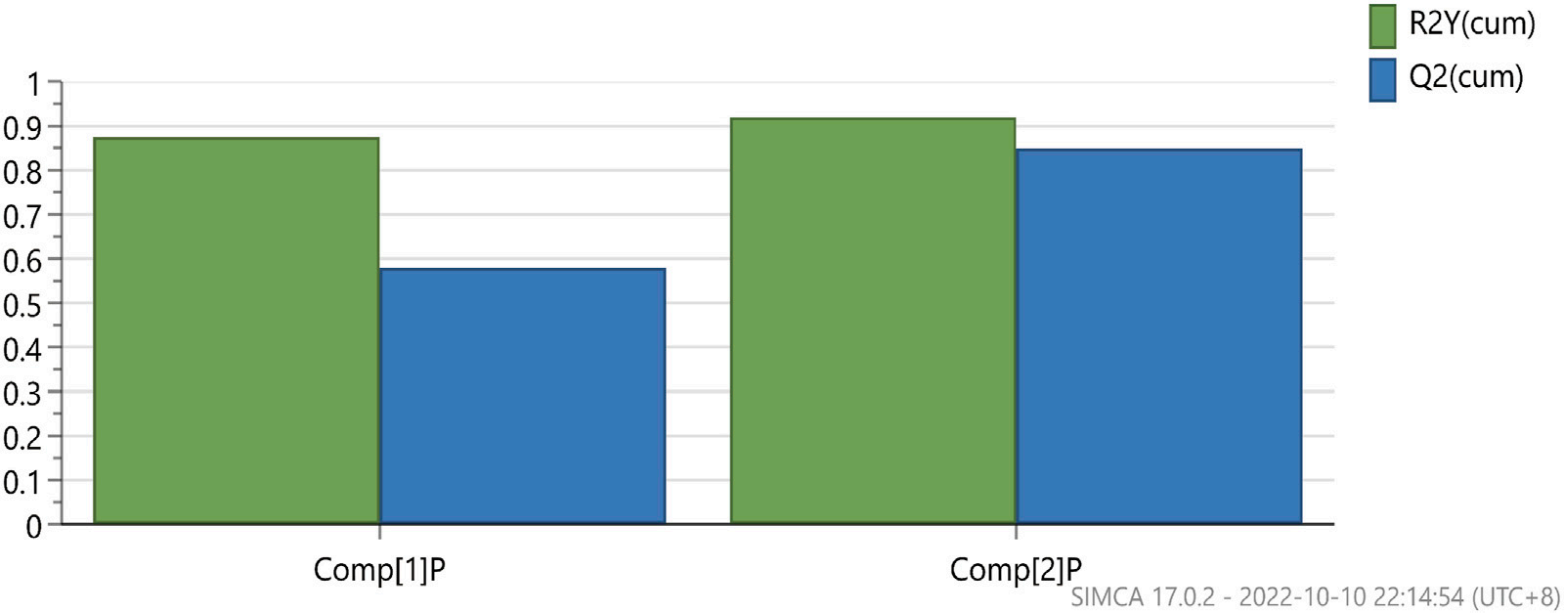
Principal component analysis of each fraction of *Asarum* essential oil.

**FIGURE 6:**
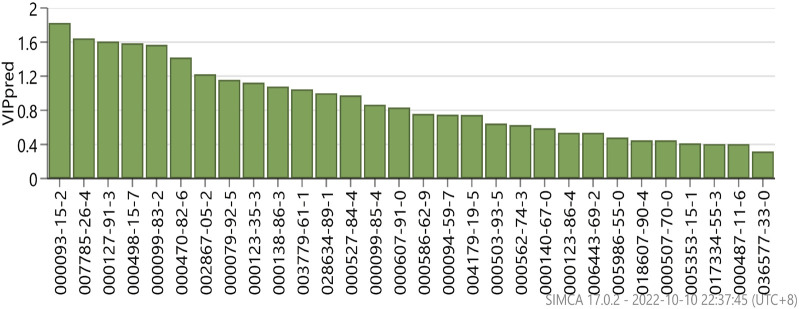
VIP values for the individual distillate compounds of *Asarum* essential oil.

Principal component analysis was used to analyze the relationship between AEO and the IL-6 and TNF-α levels via SIMCA14.1software. The results are shown in Figure 5. The data were downscaled and simplified by OPLS analysis, with Q2 (cum) of the TNF-α model being 0.832 and that of (cum) Q2 (cum) of the IL-6 model being 0.586, which showed good overall predictive ability. Further correlation analysis was performed on the two data sets to screen for compounds (Figure 6).

The correlation analysis between anti-inflammatory activity and components of AEO revealed that the anti-inflammatory activity was primarily associated with methyl eugenol, α-pinene, β-pinene, α-phellandrene, 1,8 eucalyptol, camphorene, and β-laurelene. Therefore, the strong anti-inflammatory activity of L may be related to the high relative percentage content of α-pinene and β-pinene.

### 3.6 ROS levels in PC12 cells after treatment with AEO and its different distillates

**FIGURE 7:**
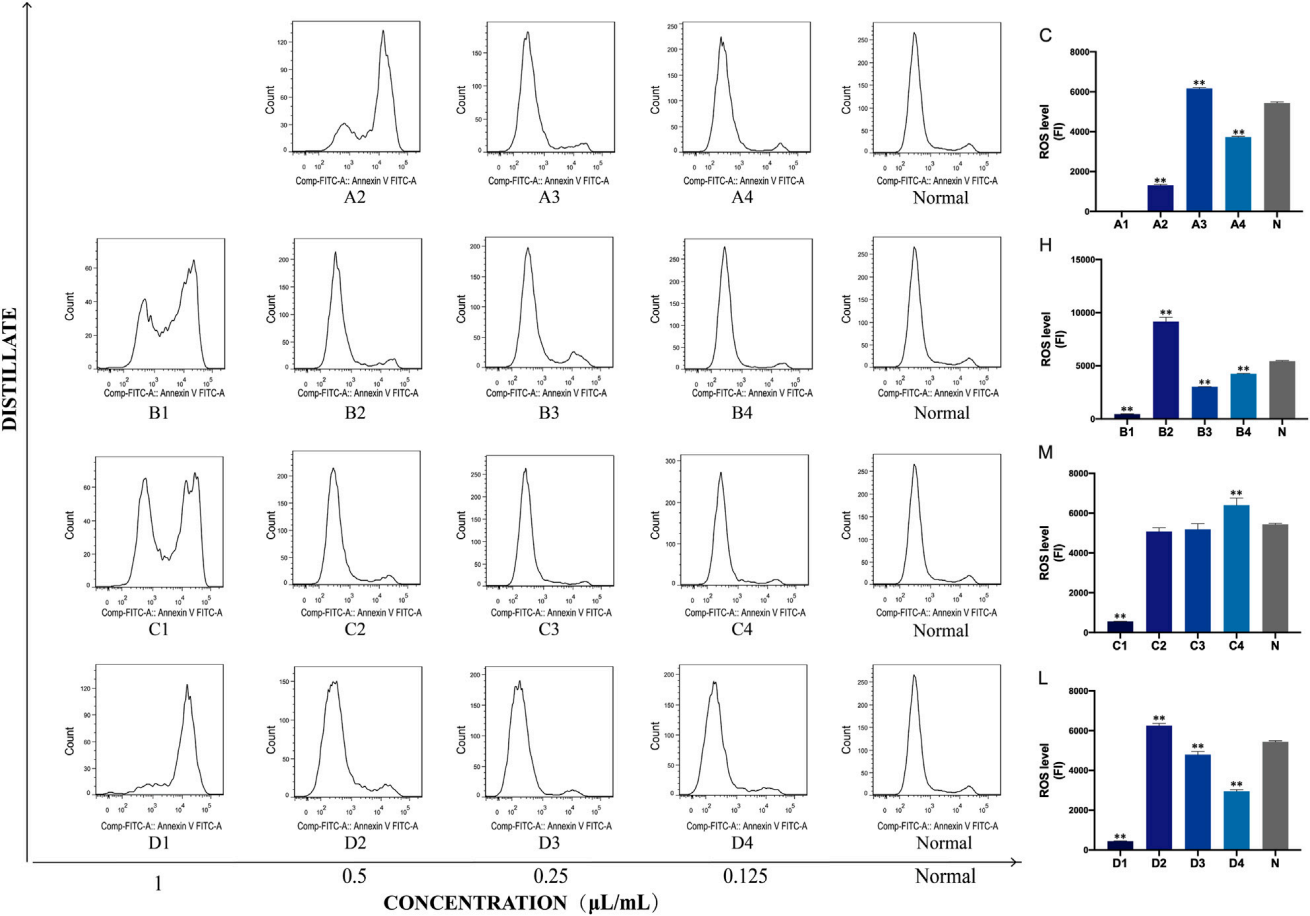
Concentrations of ROS in PC12 cells after treatment with Asarum volatile crude oil and different distillates. Concentrations of ROS in PC12 cells after treatment with 1, 0.5, 0.25, and 0.125 μL/mL of *Asarum* volatile crude oil **(C)**, heavy distillate **(H)**, medium distillate **(M)**, and light distillate **(L)**. **(A2–A4)** represent ROS levels in PC12 cells after treatment with C (**A2**: 0.5 μL/mL, **A3**: 0.25 μL/mL, **A4**: 0.125 μL/mL). **(B1–B4)** represent ROS levels in PC12 cells after treatment with H (**B1**: 1 μL/mL, **B2**: 0.5 μL/mL, **B3**: 0.25 μL/mL, **B4**: 0.125 μL/mL). **(C1–C4)** represent ROS levels in PC12 cells after treatment with M (**C1**: 1 μL/mL, **C2**: 0.5 μL/mL, **C3**: 0.25 μL/mL, **C4**: 0.125 μL/mL). **(D1–D4)** represent ROS levels in PC12 cells after treatment with L (**D1**: 1 μL/mL, **D2**: 0.5 μL/mL, **D3**: 0.25 μL/mL, **D4**: 0.125 μL/mL). The ROS levels of the **(C–L)** groups are shown on the right side, ** *p* < 0.01, compared with the normal group (N).

Next, the effect of crude oil and its distillates on intracellular ROS in PC12 cells was investigated via flow cytometry. A1 to A4 were ROS level expressions under C stimulation, B1 to B4 were under H, C1 to C4 were under M, and D1 to D4 were under L intervention. These findings from this experiment demonstrated that both the intracellular ROS levels and fluorescence intensity significantly changed after C, H, M, and L intervention compared to those of the normal group (blank group). Moreover, the fluorescence intensity tended to be normal as the drug concentration decreased. In addition, the statistical results showed that under C intervention, the fluorescence intensities of A2 (0.5 μL/mL), A3 (0.25 μL/mL), and A4 (0.125 μL/mL) were significantly changed compared with that of the normal group (*p* < 0.01). Fluorescence intensities corresponding to all four concentrations of H were statistically significant, while only C4 (0.125 μL/mL) had a significant change in M distillate (*p* < 0.01). Finally, all four concentrations (1, 0.5, 0.25, and 0.125 μL/mL) of H distillate caused significant changes in the cellular oxidative stress levels compared to the normal group (*p* < 0.01) (Figure 7).

### 3.7 Cell apoptosis of PC12 cells after treatment with AEO and its different distillates

**FIGURE 8:**
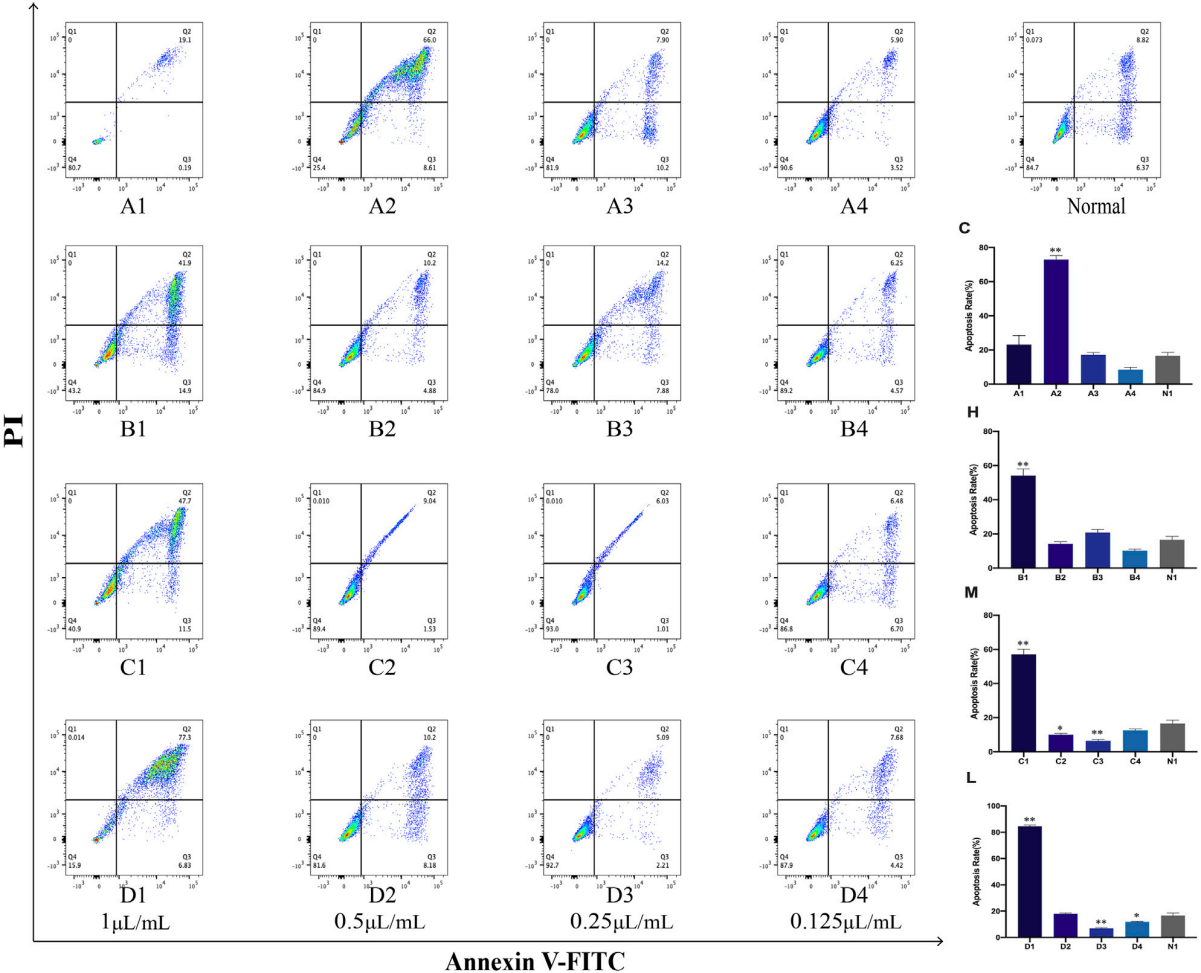
Apoptosis in PC12 cells after treatment with *Asarum* volatile crude oil and different distillates Apoptosis rates in PC12 cells were measured by flow cytometry after treatment with 1, 0.5, 0.25, and 0.125 μL/mL of *Asarum* volatile crude oil (C), heavy distillate (H), medium distillate (M), and light distillate (L). **(A1–A4)** represent apoptosis in PC12 cells after treatment with **(C)**. **(B1–B4)** represent apoptosis in PC12 cells after treatment with **(H)**. **(C1–C4)** represent apoptosis in PC12 cells after treatment with **(M)**. **(D1–D4)** represent apoptosis in PC12 cells after treatment with **(L)**. Figure on the top right represents normal PC12 cells. The lower right is the apoptosis rate in PC12 cells of the **(C, H, M, and L)** groups, *p < 0.05, **p < 0.01, compared with the normal group (N1).

The Annexin V-FITC assay kits were used to investigate the cell apoptosis rates of crude oil and distillates with PC12 cells. The results of this study showed that the high concentration (1 μL/mL) of these distillates (H, M, L) obtained after MD exhibited a higher apoptosis rates compared to the normal group (blank group). Furthermore, the apoptosis rate varied in a concentration-dependent manner. Lower concentration resulted in lower cell apoptosis rate. In addition, the statistical findings revealed that A2 (0.5 μL/mL) in distillate C and B1 (1 μL/mL) in distillate H exhibited a significant increase in apoptosis rate (*p* < 0.01, compared with that with the normal group). In M groups, the apoptosis rates of 1, 0.5, and 0.25 μL/mL were significantly different from those of the normal group. In distillate L groups, the apoptosis rates were significantly changed for the concentrations of 1, 0.25, and 0.125 μL/mL (*p* < 0.05, compared with the normal group) (Figure 8).

### 3.8 Cell morphology of PC12 cellsafter treatment with AEO and its different distillates

**FIGURE 9:**
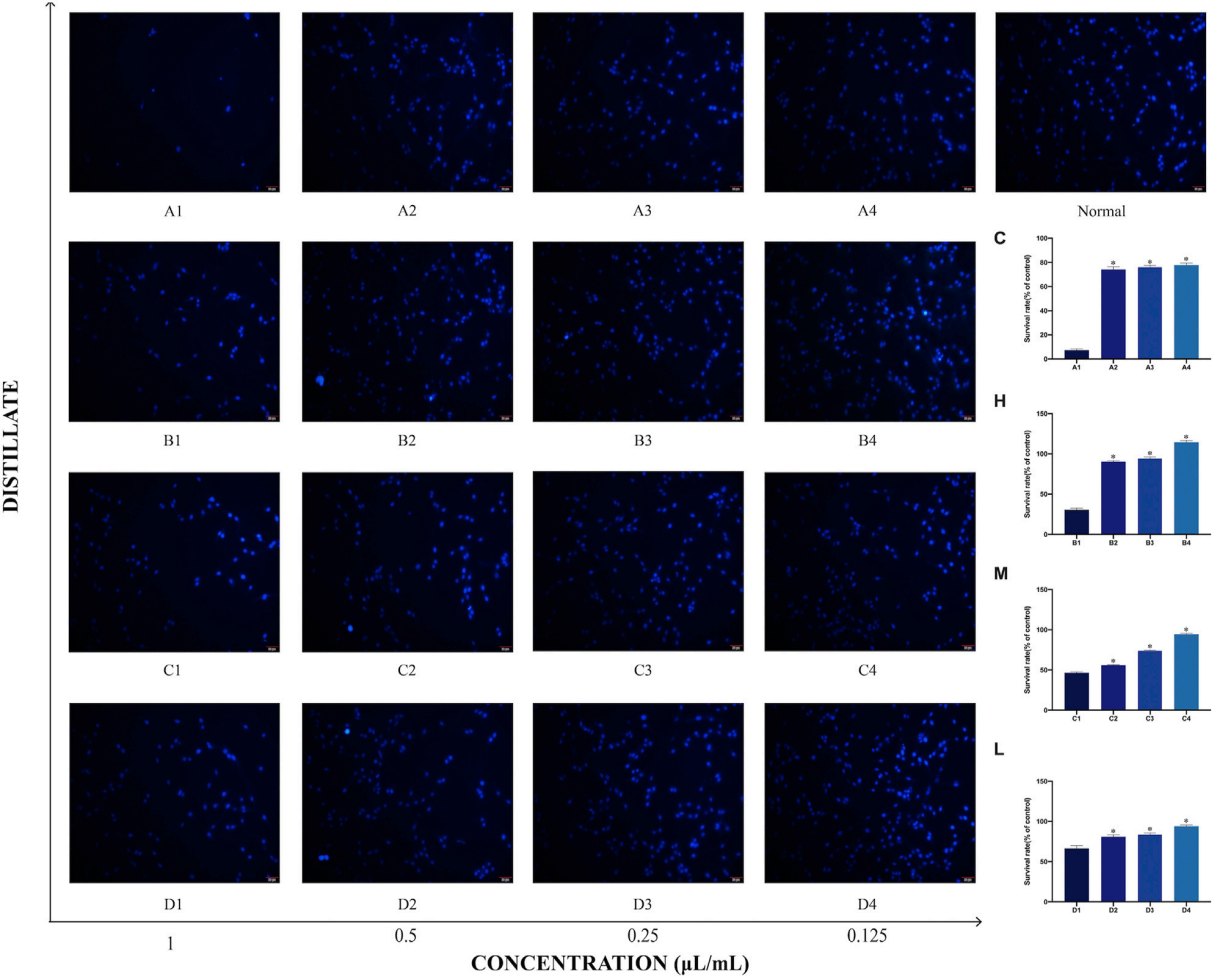
Morphology of PC12 cells after treatment with *Asarum* volatile crude oil and different distillates. The morphology of PC12 cells was measured by the DAPI staining method, scale bar: 20 μm. **(A1–A4)** represent the PC12 cells to which were added 1, 0.5, 0.25, and 0.125 μL/mL of *Asarum* volatile crude oil **(C)**, heavy distillate **(H)**, medium distillate **(M)**, and light distillate **(L)**, respectively. The top right corner shows normal PC12 cells. The survival rates of PC12 cells under different distillates are shown on the right side.

The effects of *Asarum* volatile crude oil and the different distillates on PC12 cell morphology by DAPI staining were observed. Cells in 1 μL/mL of each of C, H, M, and L (A1, B1, C1, and D1) groups demonstrated significant morphological changes with abnormal nuclei and increased apoptotic cells. However, as the concentration of the added distillate decreased, the morphology of cells in C, H, M, and L (A4, B4, C4, and D4) groups at 0.12 5 μL/mL of C, H, M, and L cells demonstrated similar morphology to that of the normal group (blank group), with intact nuclei and significantly more viable cells. These results suggested thatthat the survival rates of PC12 cells is concentration-responsively changed following treatment with AEO and its different distillates. (Figure 9).

### 3.9 Effect of AEO on the oxidase system

**FIGURE 10:**
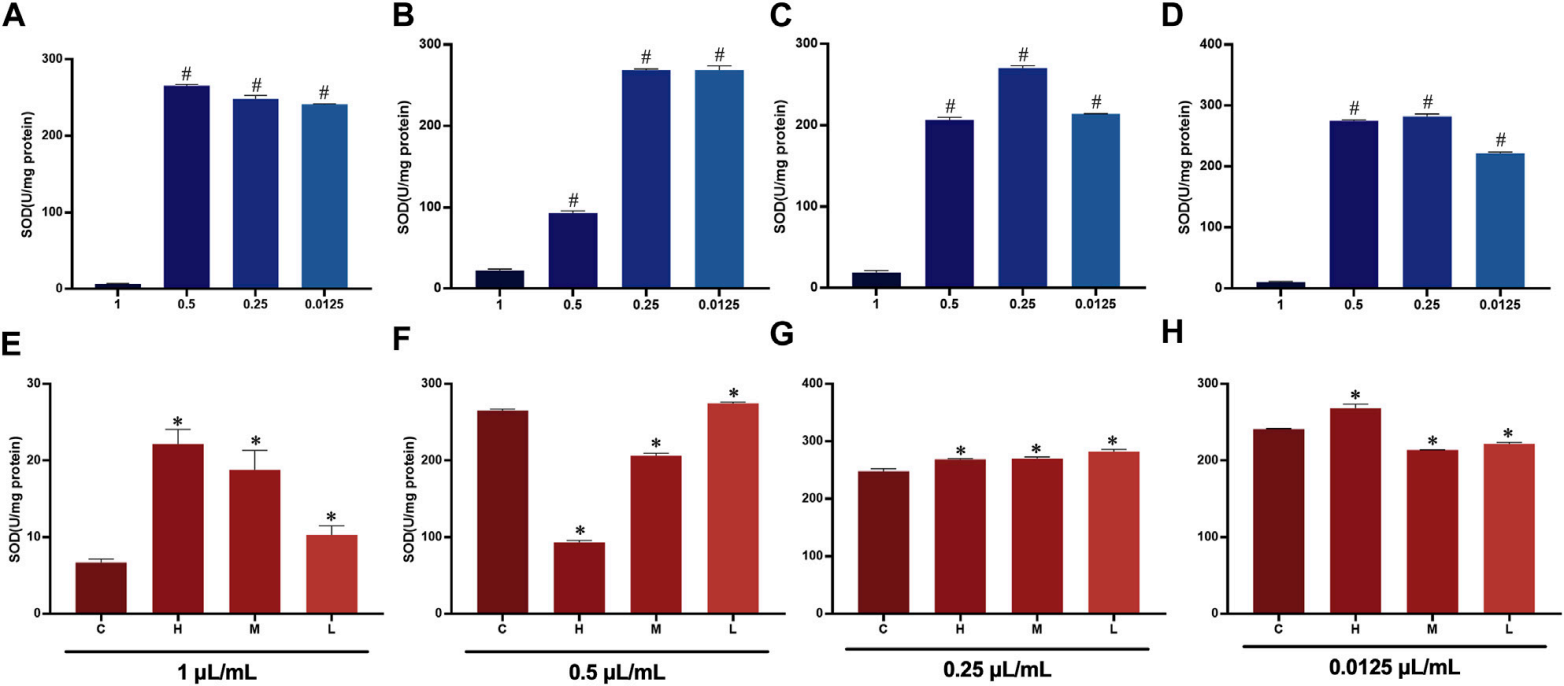
SOD expression in PC 12 cells after essential oil intervention **(A)** Expression of SOD in PC 12 cells with 0.5, 0.25, 0.125, and 0.0625 μL/mL of **(C)**. **(B)** Expression of SOD in PC 12 cells with 0.5, 0.25, 0.125, and 0.0625 μL/mL of **(H)**. **(C)** Expression of SOD in PC 12 cells with 0.5, 0.25, 0.125, and 0.0625 μL/mL of M. **(D)** Expression of SOD in PC 12 cells with 0.5, 0.25, 0.125, and 0.0625 μL/mL) of L. **(E)** Expression of SOD in PC 12 cells after treatment at 0.5 μL/mL of C, H, M, and L. **(F)** Expression of SOD in PC 12 cells after treatment at 0.25 μL/mL of C, H, M, and L. **(G)** Expression of SOD in PC 12 cells after treatment at 0.125 μL/mL of C, H, M, and L. **(H)** Expression of SOD in PC 12 cells after treatment at 0.0625 μL/mL of C, H, M, and L.

Oxidative damage can induce apoptosis. The expression levels of superoxide dismutase (SOD) in the different distillates of AEO at different concentrations are shown in. The SOD levels were significantly decreased at high concentrations of AEO, suggesting that AEO affects the oxidative stress system at high concentrations. Notably, the SOD expression of H was significantly lower than that of the other three groups (B2 < A2, C2, D2) when the concentration was 0.25 μL/mL, which may be related to the higher safrole content in H (Figure 10 **)**.

### 3.10 Effect of AEO on acute toxicity in mice

Acute toxicity experiments on mice were performed to investigate the toxicity of AEO and three AEO distillates. Mice mortality was investigated and determined after AEO administration. The results showed that the *LD*_*50*_ of C, H, and M was 1.8852, 1.9566, and 3.6741 g/Kg, respectively, and the toxicity of the M group was lower than that of the other two groups. The mortality rate of mice was only 16.67% when the administration concentration of L was 6.4185 g/Kg, indicating that the toxicity of L was significantly lower than the other distillates (Table 2).

**TABLE 2:** *LD*_*50*_ value of AEO in mice.

Groups	Dose (g/Kg)	Total (N)	Deaths (x)	Mortality (x/N, %)	*LD*_*50*_ (g/Kg)
C	1.0792	6	0	0	1.8852
	1.3489	6	0	0	
	1.6862	6	3	50	
	2.1077	6	3	50	
	2.6347	6	6	100	
H	1.0792	6	0	0	1.9566
	1.3489	6	1	16.67	
	1.6862	6	1	16.67	
	2.1077	6	3	50	
	2.6347	6	6	100	
M	2.1077	6	0	0	3.6741
	2.6347	6	1	16.67	
	3.2865	6	2	33.33	
	4.1080	6	3	50	
	5.1349	6	6	100	
L	2.6347	6	0	0	/
	3.2865	6	0	0	
	4.1080	6	0	0	
	5.1349	6	0	0	
	6.4185	6	1	16.67	

## 4 Discussion

As the primary active component of *Asarum*, the volatile oil has pharmacological effects on the nervous, immune, and cardiovascular systems (Fung et al., 2021). Despite prior research on the chemical composition and pharmacological action of AEO, notable disagreements persist regarding its main components, such as methyl eugenol, safrole, and 3,5-dimethyltoluene (Hue et al., 2022; Gu et al., 2023). Therefore, MD was used to facilitate the enrichment and separation of AEO components. Furthermore, further research was conducted to establish the relationship between toxicity, pharmacodynamics, and the main components of AEO. The results from these investigations will help elucidate the specifically effective or toxic component of the AEO and provide references for more rational and effective clinical applications of *Asarum* in the future.

Using MD, the enrichment and separation of AEO were achieved. Compared with crude oil, the content of olefin components with lower boiling points in the heavy distillate was significantly reduced, while methyl eugenol increased from 24.0203 (C) to 31.3644% (H). However, the olefin components in the middle and light distillates achieved enrichment. The ratios of α-pinene and β-pinene contents in crude oil, heavy distillate, middle distillate, and light distillate are 1:0.1:2.5:4.7 and 1:0.1:2.8:4.4, respectively, and the total amount of α-pinene and β-pinene accounts for 39.5% of the light oil distillate, which is five times more than that of crude oil. Furthermore, significant differences in the relative contents of methyl eugenol, safrole, and 3,5-dimethyl methylbenzene were observed in each distillate, with 3,5-dimethyl methylbenzene in C, H, M, and L in the ratio 1:1.1:0.8:0.2 and safrole in C, H, M, and L in the ratio 1:1.1:0.7:0.2. The ratio of methyl eugenol in C, H, M, and L was 1:1.3:0.2:0.02. The methyl eugenol content was significantly reduced in the light fraction, and the safrole content was only 18.44% of the crude oil. The difference in the composition of each distillate was influenced by the different boiling points of the components, providing a basis for further toxicological investigation.

Numerous experiments have shown that the AEO exhibits a significant anti-inflammatory effect both *in vivo* and *in vitro* (Zhang et al., 2021) (Antsyshkina et al., 2020). During inflammation, activated inflammatory cells secrete large amounts of pro-inflammatory cytokines IL-1β, IL-6, and TNF-α (Siouti and Andreakos, 2019) (Choudhury et al., 2021). Therefore, in the current study, the anti-inflammatory activity of the essential oil was chosen as the index for the evaluation of the efficacy of the essential oil. In the LPS-induced RAW264.7 cell model, LPS caused an increase in IL-6 and TNF-α levels. AEO and its fractions significantly reduced the elevation of TNF-α and IL-6 and decreased the production and release of the pro-inflammatory cytokines TNF-α and IL-6, resulting in anti-inflammatory effects. Principal component analysis showed that the monoterpene component had better anti-inflammatory activity than other components. The relative percentage of monoterpenes was higher in the light fraction than in the other fractions, so it had a better anti-inflammatory activity.

A previous study by our group demonstrated that AEO at a dose of 1.8852 g/kg inhibited the normal functioning of the central nervous system of KM mice and impaired their balance and coordination, with clinical manifestations including an intoxicated state, abnormal gait, limping, slowed movement, and the loss of appetite. These results are consistent with the reported adverse events of *Asarum*, including hepatotoxicity, nephrotoxicity, and neurologic reactions (Zhang et al., 2019). According to clinical reports, AEO could cause respiratory excitation at low doses and respiratory depression at high doses (Yan, 2022). Because of the two-way modulation of the nervous system by AEO, it is vital and significant to find the threshold between the therapeutic effect and toxicity and determine a safe therapeutic window. The acute toxicity test in mice indicated that the *LD*_*50*_ assessment confirmed that the toxicity of the crude oil was similar to that of the heavy distillate. In contrast, the toxicities of the middle and light distillates were remarkably reduced. A comparison of the components revealed that the reduction in toxicity was associated with a reduction in the contents of safrole, methyl eugenol, and 3,5-dimethyl methylbenzene.

Only a few studies are available on the mechanism of neurotoxic effects of AEO, which mainly focus on cell apoptosis, oxidative stress, and the inhibition of neuraxial growth (Hue et al., 2022). Prior evidence has indicated that mitochondrial dysfunction is a major pathogenesis of neurological diseases (Reiss et al., 2022) (Calvo-Rodriguez and Bacskai, 2021). Hence, it was investigated whether the mechanism of toxicity was related to the induction of apoptosis by the mitochondrial pathway involving ROS. PC12 cells, which are extensively applied to study neurotoxicity, are derived from the sympathetic nervous system that has stopped dividing and grows neurons when induced by nerve growth factors (Kagan et al., 2022; Xie et al., 2023). Therefore, PC12 cells were chosen to establish an *in vitro* model to explore AEO neurotoxicity in this study. According to the flow cytometric detection, biochemical methods, and the DAPI staining, it was demonstrated that AEO administration induced oxidative damage and dysfunction in the mitochondria of PC 12 cells, with significant changes in cell morphology, abnormal nuclei, and an increase in apoptotic cells. Moreover, we also noted that the apoptosis rate of PC12 cells was increased after AEO treatment, indicating that AEO could induce apoptosis in PC12 cells. In summary, the enrichment of the AEO by MD confirmed that the primary anti-inflammatory components in the AEO were methyl eugenol, α-pinene, β-pinene, α-phellandrene, 1,8 eucalyptol, camphorene, and β-myrcene, and the main toxic components were safrole, methyl eugenol and, 3,5-Dimethoxytoluene. This finding is consistent with previous literature reports. Methyl eugenol and myristicin in AEO have been reported to have hepatorenal toxicity and genotoxicity (Cao et al., 2020) (Akhlaq et al., 2022) (Carvalho et al., 2022). A study has found that safrole oxide can cause apoptosis in mouse neuronal cells by regulating the activity of ROS (Su et al., 2007a). In the meantime, safrole oxide could affect the activity of VECs, thereby inhibiting the differentiation of NSCs and inducing their apoptosis (Su et al., 2007b).

The *in vitro* findings were also validated by *in vivo* experiments. The experimental results in mice demonstrated that the light distillates of AEO exhibited low toxicity. However, the characteristic components of AEO were safrole, methyl-eugenol, and 3,5-Dimethoxytoluene, and the light distillates contained relatively low levels of the characteristic components and were not representative of AEO. Therefore, further investigations are needed to apply the MD technique to enrich the distillates containing the characteristic constituents of AEO and identify a safety window for clinical applications by studying the varying proportions of the constituents.

## 5 Conclusion

In summary, the MD technique can effectively enrich and separate essential oils. AEO exhibits good anti-inflammatory activity and considerable toxicity. The potential mechanism of neurotoxicity is related to oxidative stress and apoptosis. Most of the current methods of reducing the toxicity of *Asarum* are based on increasing the decoction time and reducing the essential oil content. However, studies have indicated that the essential oil of *Asarum* exhibits a significant anti-inflammatory effect, and reducing the essential oil content also reduces its effectiveness. Therefore, determining the safety window is essential for the clinical applications of AEO, both to exploit its medicinal properties and avoid the toxicity of the essential oil. In the current study, the MD technique was used to enrich and separate the *Asarum* essential oil and provide methods and ideas for future experiments.

Based on our previous studies, AEO has anti-inflammatory, antibacterial and analgesic effects. Through molecular distillation technology to reduce its toxicity, it is expected to be developed as a spray gel for treating oral ulcers.

## Data Availability

The raw data supporting the conclusion of this article will be made available by the authors, without undue reservation.

## References

[B1] Akhlaq S. Ara S. A. Fazil M. Ahmad B. Akram U. Haque M. (2022). Ethno pharmacology, phytochemical analysis, safety profile, prophylactic aspects, and therapeutic potential of Asarum europaeum L. in Unani medicine: An evidence-based appraisal. Phytomedicine Plus 2, 100226. 10.1016/j.phyplu.2022.100226

[B2] Antsyshkina A. M. Ars Yu.V. Bokov D. O. Pozdnyakova N. A. Prostodusheva T. V. Zaichikova S. G. (2020). The genus asarum L.: A phytochemical and ethnopharmacological review. Syst. Rev. Pharm. 11 (5), 472–502. 10.31838/srp.2020.5.66

[B4] Calvo-Rodriguez M. Bacskai B. J. (2021). Mitochondria and calcium in alzheimer’s disease: From cell signaling to neuronal cell death. Trends Neurosci. 44, 136–151. 10.1016/j.tins.2020.10.004 33160650

[B5] Cao S. Han L. Li Y. Yao S. Hou S. Ma S. (2020). Integrative transcriptomics and metabolomics analyses provide hepatotoxicity mechanisms of asarum. Exp. Ther. Med. 20, 1359–1370. 10.3892/etm.2020.8811 32742371 PMC7388312

[B6] Carvalho R. P. R. Ribeiro F. C. D. Lima T. I. Ervilha L. O. G. de Oliveira E. L. de Oliveira Faustino A. (2022). High doses of eugenol cause structural and functional damage to the rat liver. Life Sci. 304, 120696. 10.1016/j.lfs.2022.120696 35679916

[B7] Choi S. Jung M. A. Hwang Y. H. Pyun B. J. Lee J. Y. Jung D. H. (2021). Anti-allergic effects of Asarum heterotropoides on an ovalbumin-induced allergic rhinitis murine model. Biomed. Pharmacother. 141, 111944. 10.1016/j.biopha.2021.111944 34328098

[B8] Choudhury C. Mazumder R. Biswas R. Sengupta M. (2021). Cadmium exposure induces inflammation through the canonical NF-κΒ pathway in monocytes/macrophages of Channa punctatus Bloch. Fish Shellfish Immunol. 110, 116–126. 10.1016/j.fsi.2021.01.002 33453382

[B9] Dantas T. N. C. Cabral T. J. O. Dantas Neto A. A. Moura M. C. P. A. (2020). Enrichmnent of patchoulol extracted from patchouli (Pogostemon cablin) oil by molecular distillation using response surface and artificial neural network models. J. Industrial Eng. Chem. 81, 219–227. 10.1016/j.jiec.2019.09.011

[B10] Fan Y. Yang D. Huang X. Yao G. Wang W. Gao M. (2021). Pharmacokinetic study of safrole and methyl eugenol after oral administration of the essential oil extracts of asarum in rats by GC-MS. BioMed Res. Int. 2021, 6699033–6699038. 10.1155/2021/6699033 33829063 PMC8004375

[B11] Fung T. K. H. Lau B. W. M. Ngai S. P. C. Tsang H. W. H. (2021). Therapeutic effect and mechanisms of essential oils in mood disorders: Interaction between the nervous and respiratory systems. IJMS 22, 4844. 10.3390/ijms22094844 34063646 PMC8125361

[B12] Gu E. Y. Jung J. Back S. M. Lim K. H. Kim W. Min B. S. (2023). Evaluation of genotoxicity and 13-week subchronic toxicity of root of Asarum heterotropoides var. seoulense (Nakai) Kitag. J. Ethnopharmacol. 305, 116012. 10.1016/j.jep.2022.116012 36567041

[B13] Han J. M. Kim M. H. Choi L. Y. Kim G. Yang W. M. (2022). Exploring the potential effects and mechanisms of asarum sieboldii radix essential oil for treatment of asthma. Pharmaceutics 14, 558. 10.3390/pharmaceutics14030558 35335934 PMC8953372

[B14] Hou H. Li Y. Xu Z. Yu Z. Peng B. Wang C. (2023). Applications and research progress of Traditional Chinese medicine delivered via nasal administration. Biomed. Pharmacother. 157, 113933. 10.1016/j.biopha.2022.113933 36399826

[B15] Hu Z. X. Yu Z. Peng B. (2019). A literature study on the clinical dosage of foxglove before Ben Cao Zai. Jiangsu Tradit. Chin. Med. 51 (04), 69–71.

[B16] Hue H. T. T. Ky L. D. Hoang N. H. (2022). Analysis of DNA markers from Vietnamese asarum L. Species. VNU J. Sci. Nat. Sci. Technol. 38, 4. 10.25073/2588-1140/vnunst.5499

[B18] Kagan T. Stoyanova G. Lockshin R. A. Zakeri Z. (2022). Ceramide from sphingomyelin hydrolysis induces neuronal differentiation, whereas de novo ceramide synthesis and sphingomyelin hydrolysis initiate apoptosis after NGF withdrawal in PC12 Cells. Cell. Commun. Signal 20, 15. 10.1186/s12964-021-00767-2 35101031 PMC8802477

[B19] Kemprai P. Protim Mahanta B. Sut D. Barman R. Banik D. Lal M. (2020). Review on safrole: Identity shift of the ‘candy shop’ aroma to a carcinogen and deforester. Flavour Fragr. J. 35, 5–23. 10.1002/ffj.3521

[B20] Liu F. Ali T. Liu Z. (2021). Comparative transcriptomic analysis reveals the effects of drought on the biosynthesis of methyleugenol in asarum sieboldii miq. Biomolecules 11, 1233. 10.3390/biom11081233 34439899 PMC8393660

[B21] Liu G. X. Xu F. Shang M. Y. Wang X. Cai S. Q. (2020). The Relative Content and Distribution of Absorbed Volatile Organic Compounds in Rats Administered Asari Radix et Rhizoma Are Different between Powder- and Decoction-Treated Groups. Molecules 25, 4441. 10.3390/molecules25194441 32992581 PMC7582631

[B22] Liu H. Li S. Huan X. Xie Y. Xie Z. Sun Y. (2022a). The antinociceptive and anti-inflammatory potential and pharmacokinetic study of significant alkamides ingredients from Asarum Linn. J. Ethnopharmacol. 297, 115569. 10.1016/j.jep.2022.115569 35868550

[B23] Liu H. Wang C. (2022). The genus asarum: A review on phytochemistry, ethnopharmacology, toxicology and pharmacokinetics. J. Ethnopharmacol. 282, 114642. 10.1016/j.jep.2021.114642 34537281

[B24] Liu M. T. Chen X. Zhang W. (2023). Advances in the chemical composition, pharmacology and toxicology of Asarum. Chin. J. Exp. Formulation 1, 18. 10.13422/j.cnki.syfjx.20230828

[B25] Liu Y. Jiang P. Chen X. Zhang W. Shi J. (2022b). Efficacy and safety of rupatadine fumarate combined with acupoint application in allergic rhinitis complicated with diabetes. Comput. Intell. Neurosci. 2022, 6935758–6935766. 10.1155/2022/6935758 35747720 PMC9213154

[B26] Mahrous E. A. Farag M. A. (2022). Trends and applications of molecular distillation in pharmaceutical and food industries. Sep. Purif. Rev. 51, 300–317. 10.1080/15422119.2021.1924205

[B27] Maseehullah M. Zakir M. Anas M. Kazmi M. H. (2022). Ethno-pharmacology of asaroon (*Asarum europaeum* L) with special reference to unani system of medicine. J. Complementary Integr. Med. 19, 181–192. 10.1515/jcim-2021-0021 34388332

[B28] National Pharmacopoeia Commission (2020). Pharmacopoeia of the people 's Republic of China (volume one). China: China Pharmaceutical Science and Technology Press.

[B29] Nie Y. Luo Y. Luo S. Cao X. Song G. Deng C. (2022). Amphiphilic copolymers grafted on monodisperse magnetic microspheres as an efficient adsorbent for the extraction of safrole in the plasma. J. Chromatogr. A 1662, 462733. 10.1016/j.chroma.2021.462733 34902718

[B31] Reiss A. B. Ahmed S. Dayaramani C. Glass A. D. Gomolin I. H. Pinkhasov A. (2022). The role of mitochondrial dysfunction in alzheimer’s disease: A potential pathway to treatment. Exp. Gerontol. 164, 111828. 10.1016/j.exger.2022.111828 35508280

[B32] Reza Shirzad Kebria M. Rahimpour A. (2020). “Membrane distillation: Basics, advances, and applications,” in Advances in membrane Technologies. Editor Abdelrasoul A. (London, United Kingdom: Intechopen Limited). 10.5772/intechopen.86952

[B35] Siouti E. Andreakos E. (2019). The many facets of macrophages in rheumatoid arthritis. Biochem. Pharmacol. 165, 152–169. 10.1016/j.bcp.2019.03.029 30910693

[B37] Su L. Zhao B. Lv X. Wang N. Zhao J. Zhang S. (2007a). Safrole oxide induces neuronal apoptosis through inhibition of integrin beta4/SOD activity and elevation of ROS/NADPH oxidase activity. Life Sci. 80 (11), 999–1006. 10.1016/j.lfs.2006.11.041 17188719

[B38] Su L. Zhao B. Lv X. Zhao J. Zhang S. Miao J. (2007b). Safrole oxide is a useful tool for investigating the effect of apoptosis in vascular endothelial cells on neural stem cell survival and differentiation *in vitro* . Bioorg. Med. Chem. Lett. 17 (11), 3167–3171. 10.1016/j.bmcl.2007.03.032 17391961

[B41] Wang J. Zheng S. Gao Y. Shi J. Zhou Z. Ren Z. (2023). Enrichment of high‐purity nervonic acid ethyl ester from *Acer truncatum* B unge seed oil by combination method of urea inclusion and molecular distillation. *Can. J. Chem. Eng*. cjce 2023, 24875. 10.1002/cjce.24875

[B43] Wu M. Xiong Y. Han R. Dong W. Xiao C. (2020). Fumigant Toxicity and Oviposition Deterrent Activity of Volatile Constituents from Asari Radix et Rhizoma against *Phthorimaea operculella* (Lepidoptera: Gelechiidae). J. Insect Sci. 20, 32. 10.1093/jisesa/ieaa133 PMC7731873 33306098

[B44] Xie D. Deng T. Zhai Z. Sun T. Xu Y. (2023). The cellular model for alzheimer’s disease research: PC12 cells. Front. Mol. Neurosci. 15, 1016559. 10.3389/fnmol.2022.1016559 36683856 PMC9846650

[B45] Yan X. Q. (2022). Study on the concoction process and quality evaluation of Hosin. [dissertation/master’s thesis]. Jilin: Jilin Agricultural University.

[B46] Yang Q. Shen F. Zhang F. Bai X. Zhang Y. Zhang H. (2021). The combination of two natural medicines, chuanxiong and asarum: A review of the chemical constituents and pharmacological activities. J. Chem. Res. 45, 957–976. 10.1177/17475198211039130

[B48] Yao G. Ma W. Huang X. Jia Q. Shen J. Chang Y. (2020). Identification and quality evaluation of raw and processed *asarum* species using microscopy, DNA barcoding, and gas chromatography-mass spectrometry. J. Anal. Methods Chem. 2020, 2690238–2690312. 10.1155/2020/2690238 32351753 PMC7174948

[B50] Zhang H. M. Zhao X. H. Sun Z. H. Li G. C. Liu G. C. Sun L. R. (2019). Recognition of the toxicity of aristolochic acid. J. Clin. Pharm. Ther. 44, 157–162. 10.1111/jcpt.12789 30548302

[B51] Zhang Y. Li S. Liang Y. Liu R. Lv X. Zhang Q. (2021). A systematic strategy for uncovering quality marker of Asari Radix et Rhizoma on alleviating inflammation based chemometrics analysis of components. J. Chromatogr. A 1642, 461960. 10.1016/j.chroma.2021.461960 33684872

