# Supplementary material for: Effect of molecular distillation on the anti-inflammatory activity and neurotoxicity of Asarum essential oil
Source: Front Pharmacol. 2023 May 22;14:1196137. doi: 10.3389/fphar.2023.1196137 (PMC10239799; doi:10.3389/fphar.2023.1196137)
Supplement: Supplementary file 1 [file Table1.docx]

**Supplementary materials**

**Table1 Program warming conditions**

| Gradient | Heating rate (℃/min) | Temperature (℃) | Total running time (min) |
| --- | --- | --- | --- |
| Initial temperature | - | 65 | 0 |
| Gradient 1 | 3 | 80 | 5 |
| Gradient 2 | 2 | 88 | 9 |
| Gradient 3 | 0.25 | 90 | 17 |
| Gradient 4 | 0.5 | 92 | 21 |
| Gradient 5 | 5 | 122 | 27 |
| Gradient 6 | 2 | 136 | 34 |
| Gradient 7 | 20 | 300 | 43 |
